# Delayed-Interval Delivery in Dichorionic Twin Pregnancies: A Case Report of 154 Latency Days

**DOI:** 10.1055/s-0040-1701468

**Published:** 2020-01

**Authors:** Catarina Alexandra Soares de Frias, Alexandra Sofia Puga Alvarez de Faria Queirós, Helena Teresinha Fernandes Simões

**Affiliations:** 1 Department of Obstetrics and Gynecology, Hospital do Divino Espírito Santo, Ponta Delgada, São Miguel, Açores, Portugal; 2 Department of Obstetrics and Gynecology, Maternidade Dr. Alfredo da Costa, Centro Hospitalar Universitário Lisboa Central, Lisboa, Portugal

**Keywords:** delayed-interval delivery, twin pregnancy, preterm birth, maternal morbidity, neonatal morbidity, Parto diferido, Gravidez gemelar, Parto pré-termo, Morbidade materna, Morbidade neonatal

## Abstract

Premature delivery often complicates multifetal pregnancies, placing neonates at risk of serious morbidity and mortality. In select cases, preterm birth of one sibling may not require delivery of the remaining fetus(es), which may remain *in utero* for a delayed-interval delivery, consequently improving neonatal morbidity and mortality. Currently, there is no consensus on the best protocol for the optimal management of these cases. We report one case of delayed-interval delivery of a dichorionic pregnancy assisted in our center. In this case, prophylactic cerclage, tocolytic therapy and administration of broad-spectrum prophylactic antibiotics enabled delivery at 37 weeks, corresponding to 154 days of latency, which is, to our knowledge, the longest interval described in the literature. The attempt to defer the delivery of the second fetus in peri-viability is an option that should be offered to parents after counseling, providing that the clinical criteria of eligibility are fulfilled. The correct selection of candidates, combined with the correct performance of procedures, as well as fetal and maternal monitoring and early identification of complications increase the probability of success of this type of delivery.

## Resumo

O parto pré-termo espontâneo complica frequentemente as gestações multifetais, condicionando elevada morbimortalidade perinatal. Em determinados casos, o nascimento prematuro do primeiro feto pode não requerer o nascimento do(s) feto(s) restante(s), que podem permanecer *in utero*, com o objetivo de diminuir a morbidade e mortalidade neonatal. Atualmente, não existe consenso quanto à melhor atitude clínica nas situações de parto diferido. Descrevemos um caso de parto diferido de gravidez bicoriônica vigiado no nosso centro. Neste caso, a realização de cerclagem, a terapêutica tocolítica e a administração de antibioticoterapia de largo espectro permitiu o parto às 37 semanas do segundo gêmeo, o que corresponde a 154 dias de latência, que, segundo o nosso conhecimento, é o intervalo de diferimento mais longo descrito na literatura. A tentativa de diferir o parto do segundo feto na periviabilidade é uma opção que deve ser oferecida aos progenitores, após aconselhamento e desde que se cumpram os critérios clínicos de elegibilidade. A seleção correta das candidatas, em conjunto com a realização de corretos procedimentos, monitorização fetal e materna e identificação precoce de complicações aumentam a possibilidade de sucesso deste tipo de parto.

## Introduction

In the last decades, multifetal pregnancies have been increasing as a result of a higher mean maternal age and increased use of assisted reproductive technologies.^1^ ^2^ ^3^ ^4^ As a result, there is a high risk of preterm premature rupture of membranes and preterm delivery, placing neonates at risk of serious morbidity and mortality.^1^ ^2^ In twin pregnancies, delivery of the second twin generally follows the birth of the first fetus shortly thereafter. However, in selected cases, preterm birth of one sibling may not require delivery of the remaining fetus(es), who may remain *in utero* for an extended period. This event is defined as a delayed delivery of the second twin, and has been reported as a management strategy to decrease morbidity and optimize the survival of the remaining fetuses after the spontaneous preterm birth of one fetus during a multifetal gestation.^1^ ^5^ ^6^ Despite the interest in this subject, in the literature there is still a lack of an universally-accepted protocol for the optimal management of these cases.^1^ ^2^ ^3^ ^7^ ^8^ Here, we report the results of one dichorionic pregnancy with a delayed delivery of 154 days assisted at our center.

## Case Report

A 33 year-old healthy woman, with 2 previous late abortions, was followed in our institution due to a dichorionic twin pregnancy. She had two embryo transfers after a successful assisted reproductive technology (ART) cycle. She was admitted to our emergency department at 15 weeks due to regular contractions. During the pelvic examination, bulging membranes and a complete cervical dilatation was observed, with subsequent en caul delivery of the presenting fetus. After the delivery of the first twin, the uterine contractions ceased. There were no signs of chorioamnionitis. The amniotic membrane of the second twin remained intact, and ultrasonography showed a healthy remaining fetus. The parents were informed about the option of deferring delivery of the remaining fetus along with its benefits and possible complications for the mother and remaining fetus. After the parents decided to defer the delivery of the remaining fetus, cervical cultures were taken, and a McDonald cerclage was performed under general anesthesia. Tocolysis with 25 mg of indomethacin was administered 4 times a day for 2 days, and broad-spectrum antibiotics (ampicillin and gentamycin) were administered for 7 days. The mother was continuously monitored through clinical assessment and laboratory tests. No signs of infection were listed, and serial ultrasonography confirmed fetal growth and wellbeing. The cervical length was monitored weekly with transperineal ultrasonography. Due to maternal and fetal stability, the patient was discharged at the 17th week of pregnancy. A transperineal ultrasound revealed a stable cervix, with 21 mm in length. The remaining pregnancy was as expected. At the 37th week, the cervical cerclage suture was removed. And at 37 weeks and 1 day, 154 days after the delivery of the first fetus, the remaining fetus was delivered vaginally. The second baby weighed 2,980 g, and the Apgar score was 9 at the 1st minute, and 10 at the 5th minute. No maternal morbidity occurred after the delivery, and the baby girl had an uneventful neonatal course.

## Discussion

Delayed-interval delivery was first reported in the 1960s as a means of prolonging pregnancy for multifetal gestations after spontaneous delivery of the first fetus.^6^ ^9^ Since then, several case reports of asynchronous delivery have been published. To our knowledge, the published case with the longest interval between the births of both twins was also 154 days.^10^ This prolongation of the gestational period enables the reduction of premature and neonatal morbidity, and increases the survival rate of the remaining fetus.^1^ ^7^ ^10^ ^11^ Consistent with this, Van der Straeten et al^12^ reported a decrease of 13.4% in mortality with a delayed delivery of the second fetus.^2^ ^12^ It is essential that a number of conditions for deferred delivery of the second fetus are present: multifetal gestation with delivery of the first fetus before the 30th week, diamniotic pregnancy, intact membranes in the remaining gestational sac, and absence of fetal or maternal indication for delivery.^2^ ^7^ ^11^ ^13^ These inclusion criteria were all fulfilled in our case. The optimal management for a delayed-interval delivery has not yet been defined. Cerclage, tocolysis, hospitalization, and antibiotic therapy are all still controversial procedures.^1^ ^2^ ^7^ ^8^ ^9^ ^13^

The use of prophylactic cerclage is the most controversial issue among the recommended procedures.^7^ ^8^ ^11^ ^13^ ^14^ For some authors, it is a routine procedure, while for others it is recommended only if the etiology of the spontaneous delivery is cervical insuffiency.^7^ ^8^ ^11^ Zhang et al^15^ concluded that in cases of delayed-interval delivery, immediate cervical cerclage after the first delivery is associated with a significantly longer delivery interval between twins without increasing the rate of intrauterine infection.^2^ ^10^ ^15^ The median interdelivery interval was 8 and 25 days in patients without and with cervical cerclage respectively.^10^ Cerclage can minimize the exposure of the fetal membranes to vaginal bacteria, and may provide stability to the cervix.^2^ In our case, we performed a cervical cerclage and no intrauterine infection was detected. The use and duration of the tocolysis in cases of asynchronous delivery are not well established.^11^ Some authors recommend routine tocolysis after the birth of the first twin, until the contractions cease.^7^ ^13^ Others use tocolytic therapy even if there is no contractility, in cases of the performance of cerclage. In these cases, a course of 48 hours of tocolytic therapy may curb the uterine contractions precipitated by the cervical manipulation.^16^ Furthermore, studies demonstrate that the perinatal results in situations of threatened labor are not better when tocolysis are used as a maintenance therapy, or with repeated courses of tocolysis.^11^ Because of that, tocolysis with indomethacin was performed 48 hours after the prophylactic cerclage. Some authors advocate strict bed rest in the hospital until the delayed delivery. Others believe that a prolonged hospitalization is not necessary. Up to now, no management has proved to be superior to the other.^7^ ^13^ ^17^ ^18^ In the case herein reported, given the maternal and fetal stability, the patient was discharged.

We opted for broad-spectrum prophylactic antibiotics, which are routinely administered by most authors to prevent the onset of an infection.^7^ ^9^ ^11^ There is no consensus about the antibiotics of choice, the duration of the treatment, and the route of administration. However, the therapeutic scheme used in situations of preterm premature rupture of membranes could be extrapolated to cases of asynchronous delivery, because the infectious agents are similar.^11^ In addition to protecting against infections, antibiotics often have tocolytic properties.^7^ ^9^ Given the cervical stability, no steroid therapy was administered at 24 weeks. Most studies demonstrate that maternal morbidity associated with asynchronous delivery is rare.^9^ However, some authors describe a considerable incidence of serious maternal morbidity due to intrauterine sepsis and septicemia.^6^ ^7^ Careful monitoring can prevent the more serious maternal risks. We do not report any maternal complication. Neonatal survival and morbidity are primarily dependent on gestational age at birth.^6^ ^7^ Different clinical centers describe different survival rates, which range from 29% to 82%.^10^ The long-term outcome and neurological development seems to be comparable to those of children with the same gestational age.^9^ ^19^ In the case reported here, the fetal short-term outcomes were optimal, with no neonatal morbidity.

In conclusion, delayed-interval delivery is a useful and possible therapeutic option for the management of the remaining fetus, enabling the improvement of neonatal survival and decreasing morbidity.^1^ ^5^ ^6^ Selecting optimal candidates for delayed-interval delivery is fundamental, and parents should always be counseled about the potential risks and benefits of the procedure.^6^ ^11^ ^20^ ^21^ Further research in this field is needed to generate standardized management guidelines for the deferred delivery.^1^ ^2^ ^3^ ^7^ ^8^ In the case herein reported, the performance of prophylactic cerclage, a short course of tocolytic therapy, and the administration of broad-spectrum prophylactic antibiotics enabled the delivery of the second fetus at 37 weeks, corresponding to 154 days of latency.
